# Draft Genome of a Blister Beetle *Mylabris aulica*

**DOI:** 10.3389/fgene.2019.01281

**Published:** 2020-01-08

**Authors:** De-Long Guan, Xiao-Qian Hao, Da Mi, Jiong Peng, Yuan Li, Juan-Ying Xie, Huateng Huang, Sheng-Quan Xu

**Affiliations:** ^1^ College of Life Sciences, Shaanxi Normal University, Xi’an, China; ^2^ NextOmics Biosciences Institute, Wuhan, China; ^3^ College of Computer Science, Shaanxi Normal University, Xi’an, China

**Keywords:** *Mylabris aulica*, cantharidin, genome sequencing, blister beetle evolution, comparative genomics

## Abstract

*Mylabris aulica* is a widely distributed blister beetle of the Meloidae family. It has the ability to synthesize a potent defensive secretion that includes cantharidin, a toxic compound used to treat many major illnesses. However, owing to the lack of genetic studies on cantharidin biosynthesis in *M. aulica*, the commercial use of this species is less extensive than that of other blister beetle species in China. This study reports a draft assembly and possible genes and pathways related to cantharidin biosynthesis for the *M. aulica* blister beetle using nanopore sequencing data. The draft genome assembly size was 288.5 Mb with a 467.8 Kb N50, and a repeat content of 50.62%. An integrated gene finding pipeline performed for assembly obtained 16,500 protein coding genes. Benchmarking universal single-copy orthologs assessment showed that this gene set included 94.4% complete Insecta universal single-copy orthologs. Over 99% of these genes were assigned functional annotations in the gene ontology, Kyoto Encyclopedia of Genes and Genomes, or Genbank non-redundant databases. Comparative genomic analysis showed that the completeness and continuity of our assembly was better than those of *Hycleus cichorii* and *Hycleus phaleratus* blister beetle genomes. The analysis of homologous orthologous genes and inference from evolutionary history imply that the *Mylabris* and *Hycleus* genera are genetically close, have a similar genetic background, and have differentiated within one million years. This *M. aulica* genome assembly provides a valuable resource for future blister beetle studies and will contribute to cantharidin biosynthesis.

## Introduction

Cantharidin (C_10_H_12_O_4_, CTD) is a monoterpene chemical compound produced by some species of the Meloidae family from Coleoptera, commonly known as blister beetles or Spanish flies (Crowson, 1970; Carrel et al., 1993; Karras et al., 1996; Tagwireyi et al., 2000; Jiang et al., 2019). CTD is anti-inflammatory, antiviral, and increases immune-regulating activities (Moed et al., 2001); therefore, it has been widely used to treat a variety of diseases including skin-related diseases (furuncles and piles) (Su et al., 2016), tuberculous scrofuloderma (Silverberg et al., 2000; Moed et al., 2001; Richard et al., 2014) and erectile dysfunction (Wang, 1989; Liu and Chen, 2009). New research has also reported that CTD and its derivatives inhibit the proliferation of many types of cancers including pancreatic (Li et al., 2010), liver (Zhang et al., 2014), lung (Luan et al., 2010), and breast cancer (Dorn et al., 2009; Li et al., 2017). CTD has grown in popularity as an alternative to anticancer drugs, and increasing attention is being paid to methods for acquiring this chemical owing to its promising prospects as an anti-tumor agent (Kadioglu et al., 2014).

As a natural source for obtaining CTD, the CTD biosynthesis mechanism in certain blister beetles has been the focus of multiple studies (Agranoff et al., 1960; McCormick et al., 1986; Lu et al., 2016). The partial genomes of the most commercially important species in Chinese traditional medicine, *Hycleus cichorii* and *Hycleus phaleratus*, have been determined to provide the genetic background of blister beetles (Jiang et al., 2017). Moreover, according to the transcriptome analysis of *Mylabris cichorii*, another blister beetle also widely used in clinical treatment, the mevalonate (MVA) pathway and juvenile hormone biosynthesis may contribute to CTD biosynthesis (Huang et al., 2016). Although these findings provide a references for the experimental study of biosynthetic pathway mechanisms in different meloid beetles (Yin and Jin, 2010; Lu et al., 2016; Huang et al., 2016), the whole genome data required to study the genetic basis of the entire process remains unavailable (Wu et al., 2018). In addition, although many genes involved in CTD biosynthesis have been identified, new genes may still be identified using a newly obtained genome (Yin and Jin, 2010; Huang et al., 2016; Jiang et al., 2017). Therefore, utilizing multiple genomes from blister beetle species able to produce CTD will enable comparative genomic research to provide better understanding of the entire production mechanism. A whole gene set is helpful to accelerate research and provide useful background information on the systematic and evolutionary processes of CTD producing species. Additional genomes from the Meloidae family will enable researchers to trace the development of CTD biosynthesis. Generation of a genome from the *Mylabris* genera will provide a reference for other related species and value for further physiological and evolutionary research experiments. *Mylabris aulica* is a commonly distributed blister beetle with a large population in inner-Mongolia, China, and the same ability to produce CTD as other blister beetles; therefore, this species has great research potential as a prospect to obtain natural CTD. This species has been utilized for medical purposes, but not for commercial trade (Carrel et al., 1986).

Here, we report the first draft genome assembly of *M. aulica* (NCBI: txid1914941) using long reads. This study obtained and assembled the *M. aulica* genome sequence, then annotated the identified genes to further explore the characteristics of the *M. aulica* genome while paying particular attention to CTD biosynthesis. By combining time-tree construction and orthologous analyses with related species, we tentatively explored the time frame when CTD biosynthesis likely appeared in these species. To learn more about the genes related to CTD biosynthesis, we analyzed the similarity and differences of genes in *M. aulica* and related species. Knowledge of the genetic background of this and similar species will not only contribute to the study and usage of blister beetles, but also aid in supplying the increasing demand for naturally derived CTD, thereby lowering prices for this medicinal material.

## Materials and Methods

### Sample Collection and Sequencing

Twenty-one adult *M. aulica* (NCBI txid1914941) beetles were collected from Hailaer, Inner-Mongolia Province, China in August 2018. Genomic DNA was extracted from each individual male beetle using DNAeasy Tissue Kits (Qiagen, Halden, Germany). With the retrieved genomic DNA, two DNA libraries of different insert sizes were constructed and the Illumina X-ten (Illumina HiSeq X-Ten, San Diego, California) and Nanopore promethION (Oxford Nanopore Technologies) (Wouter et al., 2019) platforms were used for DNA sequencing. The short-read Illumina sequencing library was obtained by performing g-TUBE fragmentation, repair, adaptor connection, digestion with exonuclease, and recycling approximately 350 bp sequences using approximately 1.5 μg DNA according to standard sequencing kit protocol (NEBNext Ultra DNA Library Prep Kit for Illumina). The long-read Nanopore sequencing library was constructed using 5 μg DNA and the SQK-LSK109 sequencing preparation kit (Ligation Sequencing Kit). The retrieved library had a mean DNA fragment length of approximately 20 kb.

### Genome Assembly, Polishing, and Completeness Assessment

After sequencing, a strict quality control on the raw Illumina and Nanopore sequencing data was performed using Trimmomatic v0.39 (Bolger et al., 2014) and Nanofilt v2.3.0 (De Coster et al., 2018), respectively. Reads with low quality (Q30 < 90%) or those that contained more than 5% unknown bases were removed. Environmental microbe contamination was removed by deleting sequences that provided hits in the GenBank env_nt database (ftp://ftp.ncbi.nlm.nih.gov/blast/db/). Before assembly, a k-mer based analysis was performed to estimate genome size using GCE (genome characteristics estimation) (Liu et al., 2009; Manekar and Sathe, 2018) using all the short-read DNA sequences. The estimated size of the genome guided further assembly by helping with software parameter adjustments. A pipeline integrating CANU (Koren et al., 2017) and MECAT (Xiao et al., 2017) was then used to conduct the assembly using genome sequencing data with default parameters. CANU was used to generate more accurate self-corrected reads with a corrected error rate equal to 0.050. MECAT was used to generate contigs. To improve assembly accuracy, the generated Nanopore sequenced data assembly was polished using Pilon (Walker et al., 2014) with next generation data. The Nanopore sequenced data was mapped back to the assembly with Minimap2 (Li, 2018) to check the correctness. Whole genome completeness was assessed using BUSCO (benchmarking universal single-copy orthologs) v3 (Simao et al., 2015).

### Repetitive Elements Determination

Combined specific repeated sequence database and repetitive element identification was performed using the Repeatmasker (Chen, 2004) and Repeatmodeler (Castoe et al., 2011) pipelines. First, a novel library for repetitive elements was constructed using Repeatmodeler. Then, this novel library was merged into a database with all the known repetitive elements from the Insecta phylum. Then, using this database, a thorough search for repetitive elements was conducted in Repeatmasker. As a supplemental search, other types of repetitive sequences were also identified using LTR FINDER (Xu and Wang, 2007), MISA (http://pgrc.ipk-gatersleben.de/misa/) (Beier et al., 2017), and RepeatScout (Price et al., 2005) Finally, the results generated from the previous search were merged with these supplemental repetitive sequences and masked from the genome. The masked repeat sequences were further classified according to their different types (**Table S1**).

### Gene Finding and Annotation

A high-quality gene set was obtained using the combined methods of *ab initio* and homology-based predictions. The RNA-seq (Huang et al., 2016) data of *M. cichorii* was used to search for the best gene model in PASA (Haas et al., 2003). Then, this model was used in Augustus (Burge and Karlin, 1997) and SNAP (http://korflab.ucdavis.edu/) (Goodswen et al., 2012) for *ab initio* prediction. For the homology-based method, we downloaded the gene sets of *H. cichorii* (Wu et al., 2018), *H. phaleratus*, *Aethina tumida* (Evans et al., 2018), *Anoplophora glabripennis* (Mckenna et al., 2016), *Tribolium castaneum* (Richards et al., 2008), *Dendroctonus ponderosae* (Keeling et al., 2013), *Leptinotarsa decemlineata* (Schoville et al., 2018), *Diabrotica virgifera virgifera* (Coates et al., 2012), and *Bombyx mori* (Duan et al., 2010) from the GenBank database. These homologous protein sequences were concatenated and imported into GeneWise (Trapnell et al., 2012) to search for genes. The pseudogenes were filtered by determining whether they could be correctly translated and had mature stop codons. In addition, a concatenated *ab initio* and homology-based gene prediction pipeline identified genes using MAKER (Campbell et al., 2014) (http://www.yandell-lab.org/software/maker.html) software. Finally, all gene prediction results were merged to yield a non-redundant reference gene set using Evidence Modeler (Haas et al., 2008) (**Table S2**). The protein sequences were extracted and subsequently put into functional annotations (**Data Sheet 1**).

### Functional Annotation of Protein-Coding Genes

Functional annotation was performed by NCBI BLAST (ftp://ftp.ncbi.nlm.nih.gov/blast/db/) (Lobo, 2012) to query the predicted gene sequences to functional databases such as NR (Li et al., 2013), KEGG (Kyoto Encyclopedia of Genes and Genomes (Keller et al., 2011), and Swiss-prot (UniProt Consortium, 2010). The Pfam database was used to annotate proteins using HMMER (Birney et al., 2004). Gene ontology (Walker et al., 2014) terms were obtained using Blast2GO (Pertea et al., 2015). Metabolic pathways of these genes were assigned using the KAAS (Moriya et al., 2007) tool provided by the KEGG database. All of these annotations can be found in **Table S3**.

### Orthologous Analysis and Time-Tree Reconstruction

Gene families were identified using OrthoFinder software (Rice et al., 2000). The protein sequences of *H. cichorii*, *H. phaleratus*, *A. tumida*, *A. glabripennis*, *T. castaneum*, *D. ponderosae*, *L. decemlineata*, *D. virgifera virgifera*, and *B. mori* were used for all-2-all comparisons to search for orthologous gene families (**Table S4**). The expansion and constriction of the orthologous gene families were determined and counted (**Table S5**). Then, the coding sequences of the single-copy genes in the 10 species were identified, extracted, and aligned using the MAFFT program (Katoh, 2002) to construct one super-matrix. The BEAST program (Yang and Rannala, 2006) was used to build the phylogenetic tree and estimate divergence time. The calibration time selection was based on two nodes: *D. ponderosae* and *A. glabripennis* [150.3–220.3 mya] (http://www.timetree.org/), and *H. cichorii* and *H. phaleratus* [9.8–44.8 mya] (Wu et al., 2018). The BEAST program parameters were as follows: GTR substitution model, Gamma & Invariant model in sites with 4-fold Gamma categories, Uncorrelated lognormal clock model, Yule process tree prior. The MCMC chain was set to 10 million generations.

## Results and Discussion

### Genome Assembly and Completeness

The *M. aulica* genome assembly was 288.5 Mb in size with a scaffold N50 length of 467.8 kb and an L50 value of 44. The repeat sequence in the assembly was 153.1 Mb and occupied 50.62% of the total length. All of these parameters were greatly improved compared to those reported for the *H. cichorii* and *H. phaleratus* genomes (N50 values of 79.3 kb and 56.1 kb and identified repetitive content of 22.73% and 13.47%, respectively) (**Table 1**). It is obvious that the Nanopore long-read sequencing technology used in this study has exponentially improved assembly connectivity, proving that it is a better strategy for obtaining highly qualified genomes.

**Table 1:** Comparison among three blister beetles' genome assemblies.

	*Hycleus cichorii*	*Hycleus phaleratus*	*Mylabris aulica*
Estimated Genome size	269.9	307.9	318.4
Assembled genome size (Mb)	111.77	106.7	288.5
Scaffold N50 (Kb)	79.3	56.1	467.8
Repeat content (% of genome)	22.73	13.47	50.62
Gene number	13813	13725	13050

**Figure 1:**
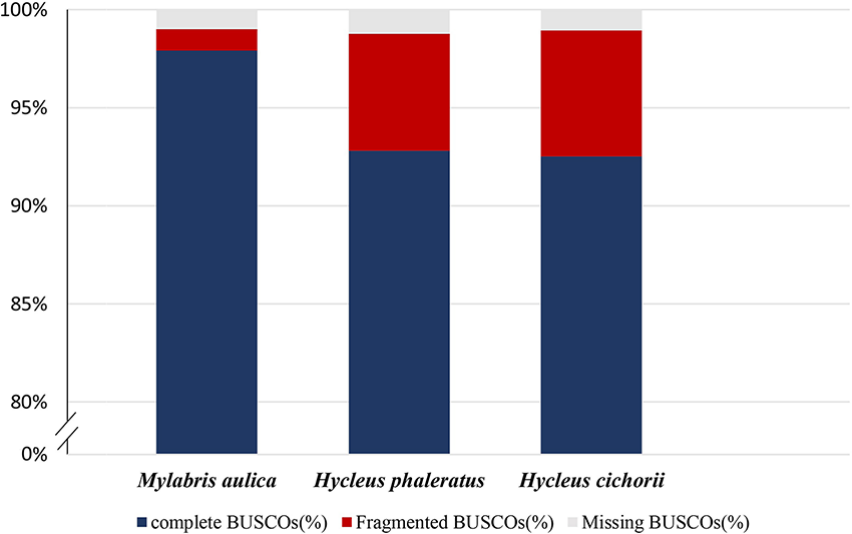
Summarized benchmarks in the benchmarking universal single-copy orthologs (BUSCO) assessment for *M. aulica*, *H. cichorii,* and *H. phaleratus* genomes. These estimations used 42 species from Insecta as the database and 1,658 BUSCOs were searched. The orange, blue, and green sections of each bar represent complete percentage, fragmented percentage, and missing percentage, respectively.

Evaluation of assembly completeness and correctness showed that the *M. aulica* assembly spanned 91% of the estimated genome size (318.4 Mb), which proves that it is highly complete with few missing sequences. Assembly error rate was estimated by mapping the genome sequencing data back to the assembly. The results showed a re-mapping coverage of 101.4× (30.41 Gb of data), and an extremely low error rate (0.09%) that can be ignored. BUSCO analysis conducted on the assembly to check coding region completeness showed that 97.9% and 1.1% of the 1658 Insecta BUSCO groups were found, and only approximately 1% were missing. This indicated that we have reconstructed almost all of the coding regions. Furthermore, comparison of the *H. cichorii* and *H. phaleratus* assembly BUSCO assessment results with that of *M. aulica* (**Figure 1**) suggests that not only the connectivity, but the completion for the obtained *M. aulica* genome assembly is superior. The assembly yielded by Nanopore sequencing shows that this approach is advantageous in all respects.

### Genetic Background of *M. aulica*

Using the obtained high-quality assembly, we explored the basic genetic background of *M. aulica*. First, we identified the gene set embedded in this assembly and retrieved 16,500 genes. The average length of these genes was 5,940.82 bp and each of them contained about 6.62 exons. The average length of coding domains and exons were 1,958.63 bp and 295.72 bp, respectively. All these parameters were comparable to those of the *H. cichorii* and *H. phaleratus* assemblies. More genes were identified in the *M. aulica* genome assembly, which confirms our previous finding that our assembly has better continuity and completion than other available assemblies and could serve as the most complete genetic research material for blister beetles.

**Figure 2:**
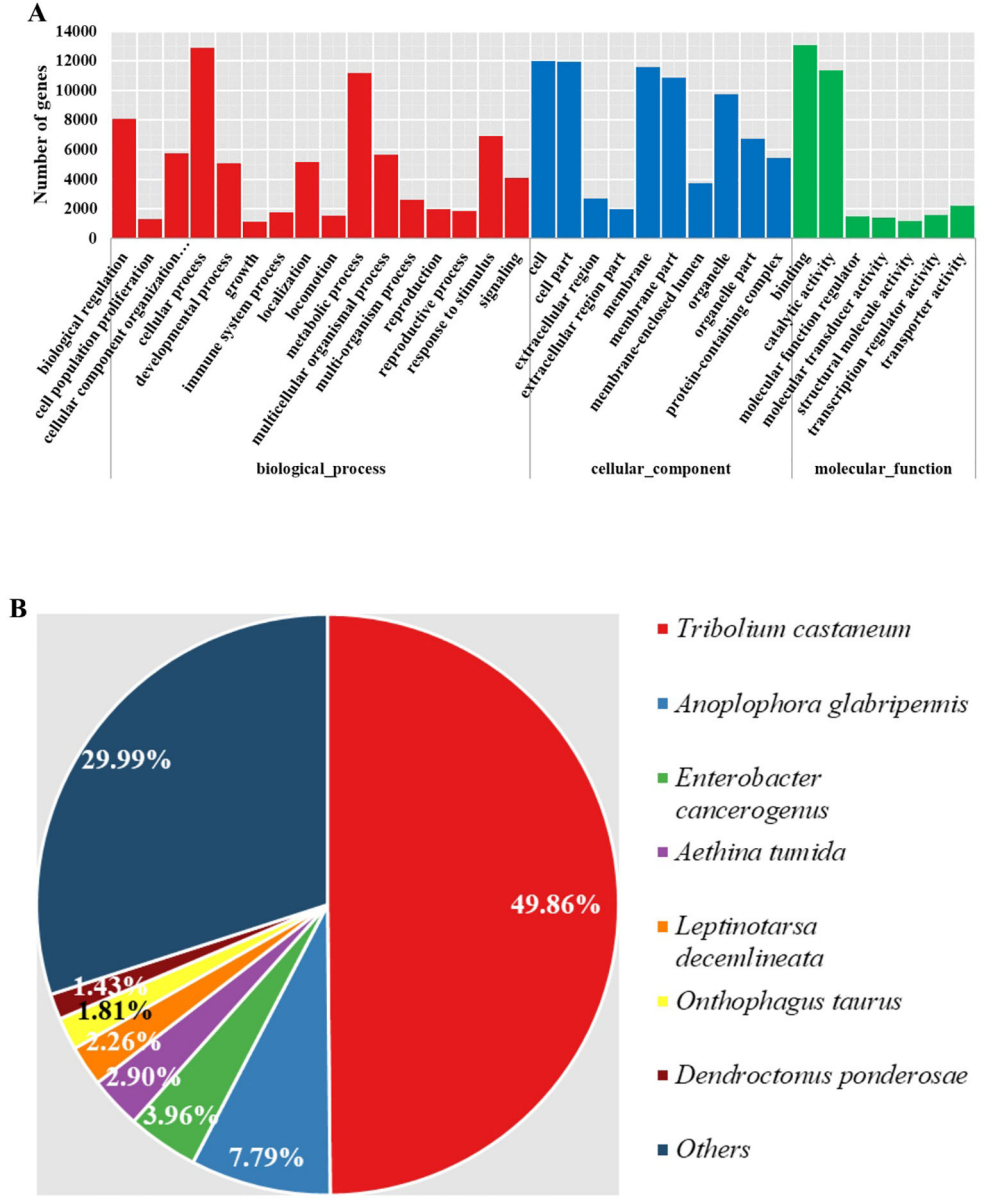
Summarized GO terms and source species contents of NR annotations. **(A)** The red, blue, and green bars represent biological process, cellular component, and molecular function terms, respectively. **(B)** Pie chart showing the different source species content in NR annotations.

Gene function retrieval from multiple databases showed that almost all genes (16,444 of 16,500, 99.7%) could be annotated. Among them, 15,579 genes could be assigned to at least one GO term, and 6269 could be assigned to different KEGG pathways. Additionally, 15,153, 13,445, and 16,131 of the genes could be annotated using Pfam (El-Gebali et al., 2018), Swiss-prot (Boutet et al., 2016), and the Genbank NR databases, respectively. The functional traits of all genes were further explored according to their annotations. The summarized GO terms prevalent in most insects and responsible most basic life activities were present and essential in *M. aulica* (**Figure 2A**). There were no specialized functional basis characters in *M. aulica*. Further, as we classified the source species of the NR annotations, we found that 79.85% of the genes resembled sequences in *T. castaneum* and *A. glabripennis* genomes, which are not blister beetles (**Figure 2B**). This result indicates that the functional basis for *M. aulica* is not largely deviated from that of common beetles, and that the genes contributing to specialized CTD synthesis only comprise a small portion of the whole genome.

### Comparative Genomic Analysis Based on Ortholog Gene Families

**Figure 3:**
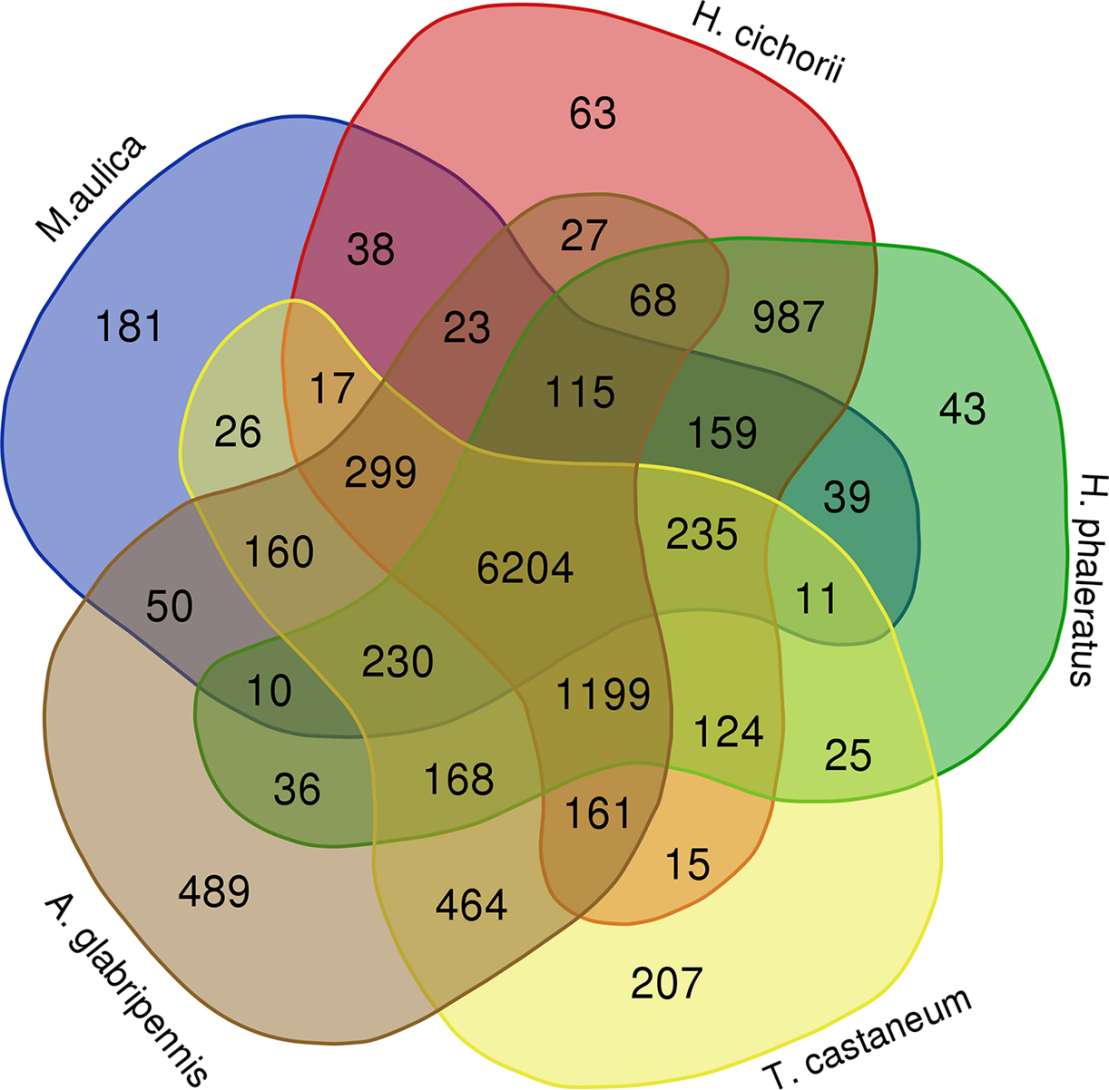
The Venn map shows the distribution pattern of ortholog gene families in *M. aulica*, *H. cichorii*, *H. phaleratus*, *T. castaneum*, and *A. glabripennis*.

Identification and analysis of ortholog gene families is the foundation of comparative genomics that enables a better understanding of the evolution of traits among different species. Overall, we identified 12,726 ortholog gene families in *M. aulica* and nine related species. Among them, 6,204 members including 48.75% of total gene families were commonly shared in *M. aulica*, *H. cichorii*, *H. phaleratus*, *T. castaneum*, and *A. glabripennis* (**Figure 3**). The results showed that these species have broad genetic connections and are suitable for phylogenetic analysis and divergence time estimation.

**Figure 4:**
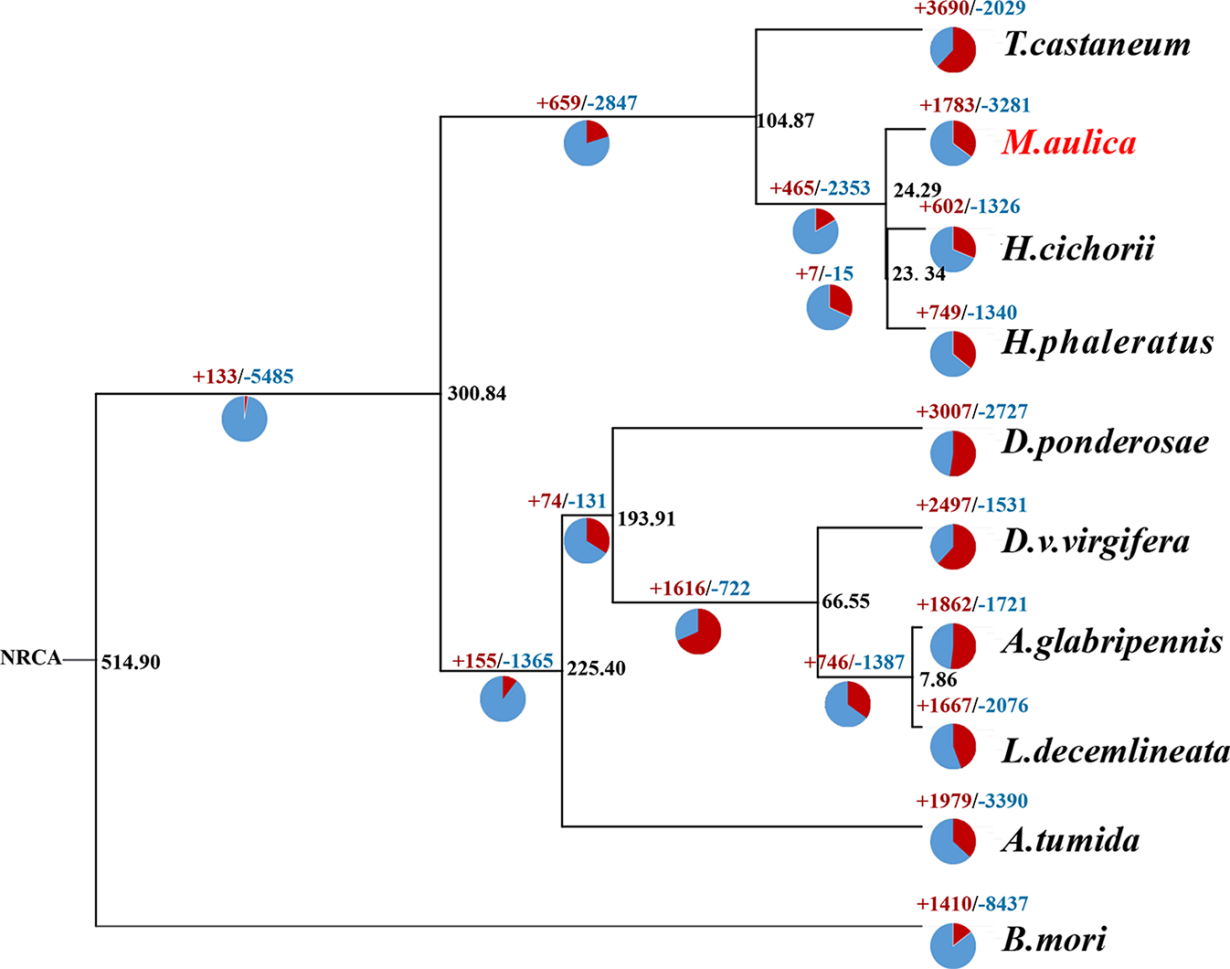
Maximum-likelihood tree of 10 selected insect species. Blue represents gene families with a decreased percentage, while red represents those with an expansion percentage. The black number on the node represents the average divergence time.

Thus, using the 435 identified single copy ortholog genes, a creditable phylogenetic tree was created (**Figure 4**). Based on this tree, we found that *M. aulica*, *H. cichorii*, and *H. phaleratus* formed a single clade with extremely close phylogenetic positions and a short divergence time. A common ancestor for these three blister beetles was suggested. Moreover, the speciation for these species were estimated to have occurred within one million years, which suggested their hypothetical common ancestor was not much older than the establishment of their species. The metabolic and biological characters of *M. aulica* should be similar to those of *H. cichorii* and *H. phaleratus*, which were inherited from their common ancestor, and the CTD synthesis mechanism could be a part of that inheritance.

**Figure 5:**
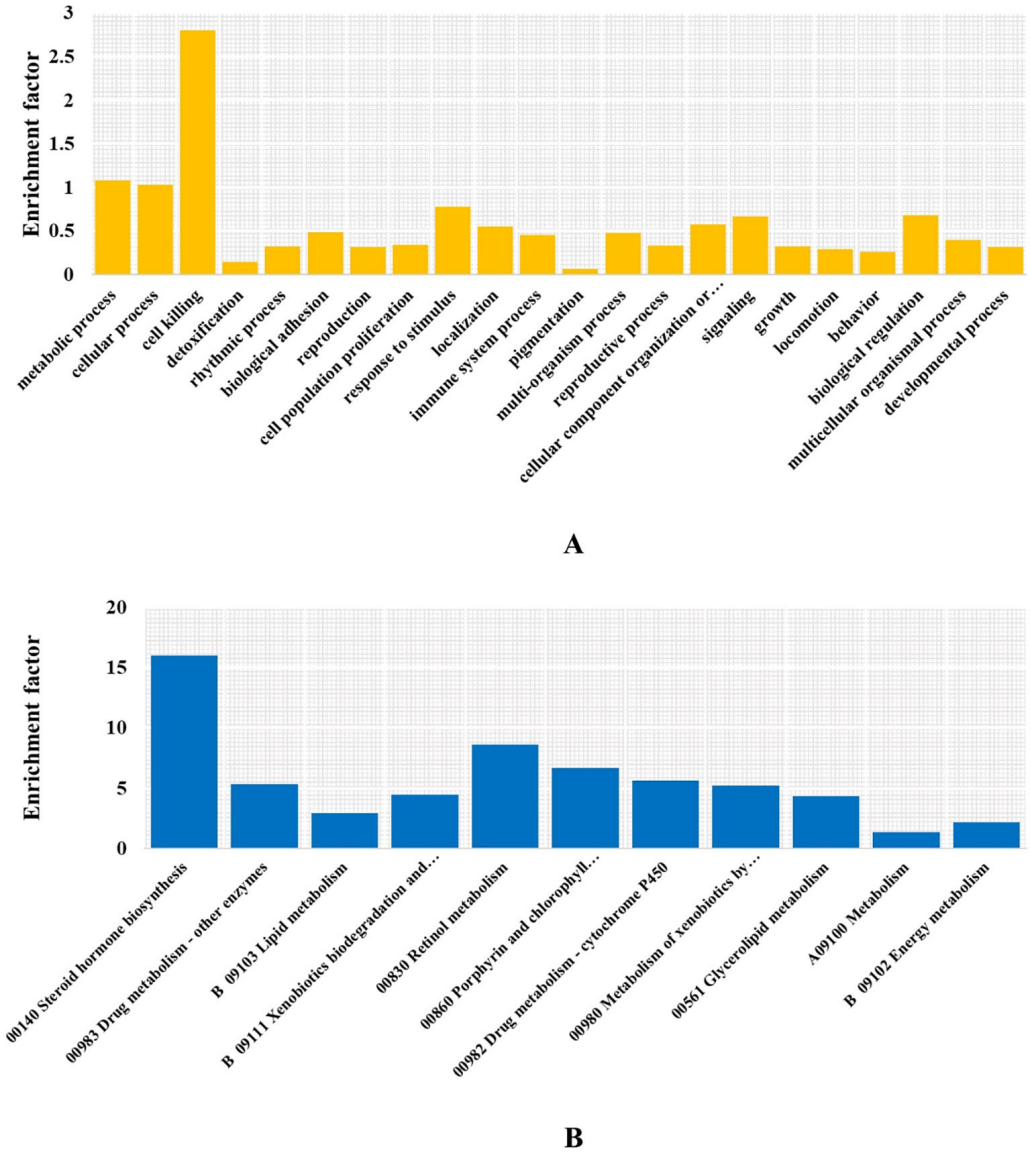
Enriched GO terms and KEGG pathways. **(A)** GO terms at level 2. **(B)** KEGG metabolism pathways.

To determine whether this CTD synthesis mechanism was solely the result of inheritance and not affected by adaption or the unique physiology formation of *M. aulica*, we identified the significantly expanded ortholog gene families within this species. The ultra-metric time tree showed that 377 of the 1,783 expanded gene families were significant. The GO and KEGG enrichment analyses of these gene families showed that none of the known genes related to CTD biosynthesis were included, and the function of the most significantly expanded genes were related to metabolic functions necessary for survival (**Figures 5A**, **B**). The CTD synthesis mechanism should have been a mature system before the species differentiation of blister beetles, which further leads to the reasonable speculation that this mechanism might be conserved in all blister beetles, except for those that have lost the ability to synthesize CTD.

### Genes Related to CTD Biosynthesis in *M. aulica*

**Figure 6:**
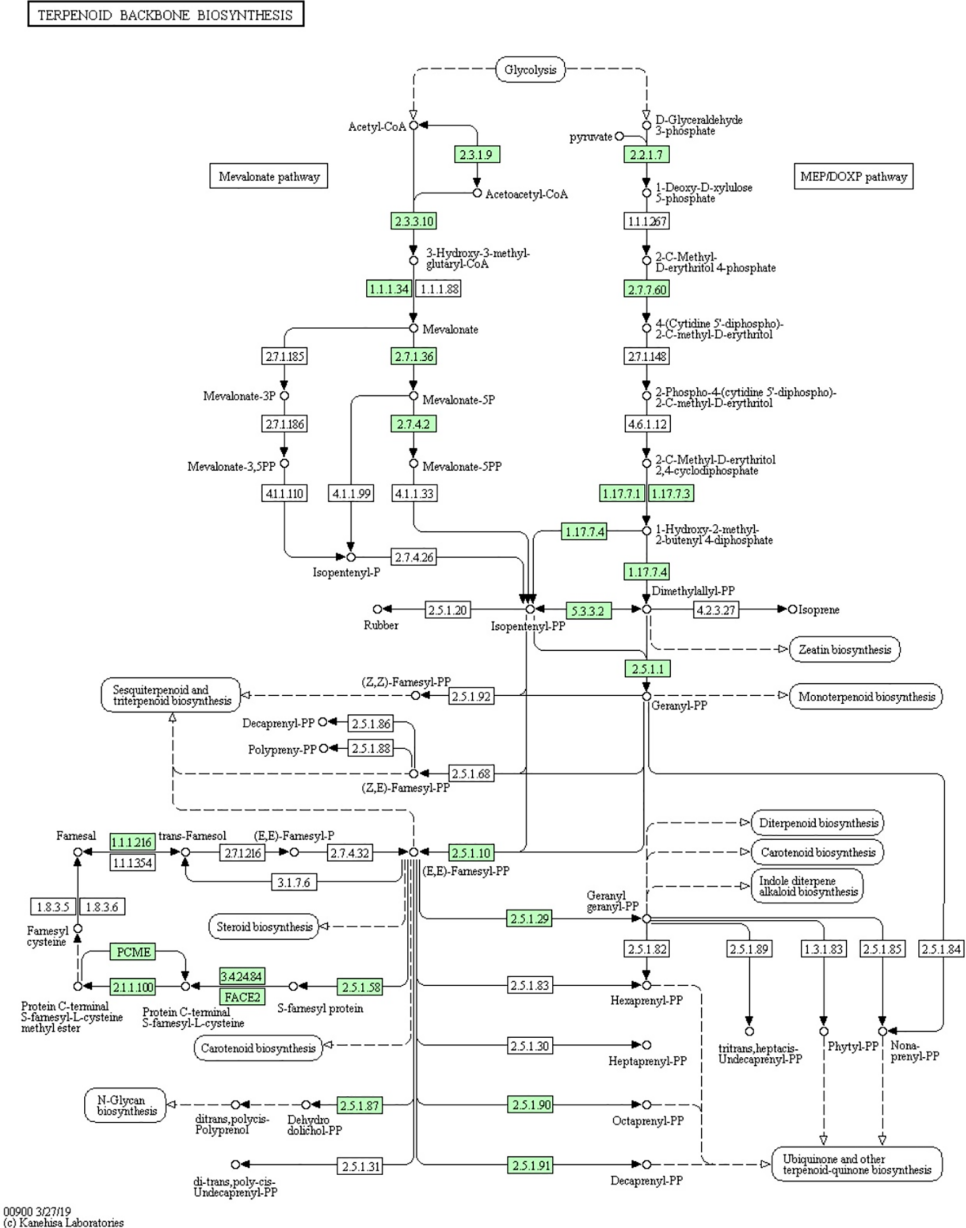
“Terpenoid backbone biosynthesis” KEGG pathway map. The highlighted green boxes represent the genes that can be found in the *M. aulica* genome.

**Figure 7:**
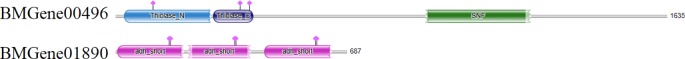
A brief gene structure map of BMGene00496and BMGene01890. Their Pfam domains were shown.

Identifying genes related to CTD biosynthesis in *M. aulica* is the major aim of our study and the primary value of our retrieved genomic data. The results provide direct evidence for the implication that the *M. aulica* has the potential to replace or fill the current medical market for blister beetles. To enable this potential, identification of the CTD biosynthesis genes in the *M. aulica* genome is required. Based on previous research, we tried to locate genes belonging to the “terpenoid backbone biosynthesis” pathway, as it is considered responsible for CTD synthesis in blister beetles (Huang et al., 2016) (**Figure 6**). In total, 30 genes in *M. aulica* matching this criterion were screened out. These genes are involved in both the MVA and MEP/DOXP pathways. These two paths synergistically participate with each other and form a complete metabolic chain in accordance with the current understanding of CTD biosynthesis in blister beetles. We then determined whether any of these 30 screened genes are unidentified genes. The results showed that all genes resembled reported sequences in the NR database, which suggested they have already been revealed by previous molecular analysis. However, two sequences (BMGene00496 and BMGene01890, **Figure 7**) were tagged as uncharacterized proteins, indicating that their biological functions remain unknown. Therefore, we performed a function search and provide their protein information for the first time. The results showed that they are enzymes that contribute to the synthesis of acetoacetyl-CoA and farnesal, respectively. The Pfam domains embedded in these two sequences show that and they might work as thiolase and short chain dehydrogenase, respectively.

## Conclusion

The ability to synthesize multiple bioactive substances, especially CTD, is an interesting and attractive biological mechanism in blister beetles; genomics research is highly significant in the study of CTD biosynthesis. However, there are few genomic studies on CTD, and complete genomic blister beetle research is even scarcer. This study provides the first *M. aulica* genome with high continuity and integrity and performs preliminary comparative genomic analyses based on this genome. Compared to previous genomic data (Huang et al., 2016), this study generated a highly-qualified genome and identified novel genes that will provide both a theoretical basis and technical support for further analyses on CTD biosynthesis or natural CTD resource mining. The continuity and completion for the *M. aulica* assembly is superior to other blister beetle genome assembly results. The findings provided by our study also provide a new understanding of the genetic background and evolution of blister beetles. Our findings imply that all blister beetles share a common ancestor, and the biological synthesis mechanism and evolution of CTD in blister beetles may just be the result of inheritance with minimal adaptive functional modification afterward. *M. aulica* has the same metabolism for CTD biosynthesis as the currently utilized species. This proves that *M. aulica* has the potential to replace the current medical use of *H. cichorii* and *H. phaleratus*. Moreover, we found 30 gene families in the “terpenoid backbone biosynthesis” pathway that contribute to CTD biosynthesis and provided functional traits for two previously uncharacterized genes that facilitate existing CTD synthesis research. These findings will pave the way for the comprehensive future study of CTD and associated biosynthesis pathways in blister beetles.

## Data Availability Statement

The datasets generated for this study can be found in the NCBI BioProject accession number PRJNA558450.

## Author Contributions

D-LG, and X-QH conceived the study and designed the experiments, they should be considered as co-first author. D-LG, JP, and DM performed the experiments. X-QH, D-LG and YL analyzed the data. X-QH and HH wrote the manuscript. S-QX and J-YX revised the manuscript. All authors read and approved the final manuscript.

## Funding

This work was financially supported by the Excellent Doctor Innovation Project of Shaanxi Normal University (S2015YB03), the Fundamental Research Funds for the Central Universities (2018CSLZ004), and the Fundamental Research Funds for the Central Universities (GK201604008; GK201702010; GK201701006). This work was supported in part by the National Natural Science Foundation of China (No. 31372250).

## Conflict of Interest

Authors DM, PJ and YL were employed by NextOmics Biosciences.

The remaining authors declare that the research was conducted in the absence of any commercial or financial relationships that could be construed as a potential conflict of interest.

## References

[B1] Beier S. Thiel T. Münch T. Scholz U. Mascher M. (2017). MISA-web: a web server for microsatellite prediction. Bioinformatics 33 (16), 2583–2585. 10.1093/bioinformatics/btx198 28398459 PMC5870701

[B2] Birney E. Clamp M. Durbin R. (2004). Genewise and genomewise. Genome Res. 14 (5), 988–995. 10.1101/gr.1865504 15123596 PMC479130

[B3] Bolger A. M. Lohse M. Usadel B. (2014). Trimmomatic: a flexible trimmer for Illumina sequence data. Bioinformatics 30 (15), 2114–2120. 10.1093/bioinformatics/btu170 24695404 PMC4103590

[B4] Boutet E. Lieberherr D. Tognolli M. Schneider M. Bansal P. Bridge A. J. (2016). “UniProtKB/Swiss-Prot, the Manually Annotated Section of the UniProt KnowledgeBase: how to use the entry view,” in Plant Bioinformatics: Methods and Protocols. Ed. Edwards D. (New York, NY: Springer New York), 23–54. 10.1007/978-1-4939-3167-5_2 26519399

[B5] Burge C. Karlin S. (1997). Prediction of complete gene structures in human genomic DNA. J. Mol. Biol. 268 (1), 78–94. 10.1006/jmbi.1997.0951 9149143

[B6] Campbell M. S. Holt C. Moore B. Yandell M. (2014). Genome annotation and curation using MAKER and MAKER-P. Curr. Protoc. in Bioinf. 48 (1), 4.11.11–14.11.39. 10.1002/0471250953.bi0411s48 PMC4286374 25501943

[B7] Carrel J. E. Doom J. P. McCormick J. P. (1986). Cantharidin biosynthesis in a blister beetle: inhibition by 6-fluoromevalonate causes chemical disarmament. Experientia 42 (7), 853–854. 10.1007/BF01941552 3732495

[B8] Carrel J. E. McCairel M. H. Slagle A. J. Doom J. P. Brill J. McCormick J. P. (1993). Cantharidin production in a blister beetle. Experientia 49 (2), 171–174. 10.1007/BF01989424 8440353

[B9] Castoe T. A. Hall K. T. Guibotsy Mboulas M. L. Gu W. de Koning A. P. J. Fox S. E. (2011). Discovery of highly divergent repeat landscapes in snake genomes using high-throughput sequencing. Genome Biol. Evol. 3,2016(2011-5-13) 3 (1), 641–653. 10.1093/gbe/evr043 PMC3157835 21572095

[B10] Chen N. (2004). Using RepeatMasker to identify repetitive elements in genomic. Curr. Protoc. Bioinformatics. 10, 1–14. 10.1002/0471250953.bi0410s05 18428725

[B11] Coates B. S. Alves A. P. Wang H. Walden K. K. O. French B. W. Miller N. J. (2012). Distribution of genes and repetitive elements in the diabrotica virgifera virgifera genome estimated using BAC sequencing. J. Biomed. Biotechnol. 5, 604076. 10.1155/2012/604076 PMC3420361 22919272

[B12] Crowson R. A. (1970). Blister Beetle Taxonomy. Nature 227 (5264), 1273–1273. 10.1038/2271273a0

[B13] De Coster W. D’Hert S. Schultz D. T. Cruts M. Van Broeckhoven C. (2018). NanoPack: visualizing and processing long-read sequencing data. Bioinformatics 34 (15), 2666–2669. 10.1093/bioinformatics/bty149 29547981 PMC6061794

[B14] Dorn D. C. Kou C. A. Png K. J. Moore M. A. S. (2009). The effect of cantharidins on leukemic stem cells. Int. J. Cancer 124 (9), 2186–2199. 10.1002/ijc.24157 19123473

[B15] Duan J. Li R. Cheng D. Fan W. Zha X. Cheng T. (2010). SilkDB v2.0: a platform for silkworm (Bombyx mori) genome biology. Nucleic Acids Res. 38, D453–D456. 10.1093/nar/gkp801 19793867 PMC2808975

[B16] El-Gebali S. Mistry J. Bateman A. Eddy S. R. Luciani A. Potter S. C. (2018). The Pfam protein families database in 2019. Nucleic Acids Res. 47 (D1), D427–D432. 10.1093/nar/gky995 PMC6324024 30357350

[B17] Evans J. D. McKenna D. Scully E. Cook S. C. Dainat B. Egekwu N. (2018). Genome of the small hive beetle (Aethina tumida, Coleoptera: Nitidulidae), a worldwide parasite of social bee colonies, provides insights into detoxification and herbivory. Gigascience 7 (12), giy138. 10.1093/gigascience/giy138 PMC6302959 30535280

[B18] Goodswen S. J. Kennedy P. J. Ellis J. T. (2012). Evaluating high-throughput ab initio gene finders to discover proteins encoded in eukaryotic pathogen genomes missed by laboratory techniques. PloS One 7 (11), e50609. 10.1371/journal.pone.0050609 23226328 PMC3511556

[B19] Haas B. J. Delcher A. L. Mount S. M. Wortman J. R. Smith R. K. Jr. Hannick L. I. (2003). Improving the Arabidopsis genome annotation using maximal transcript alignment assemblies. Nucleic Acids Res. 31 (19), 5654–5666. 10.1093/nar/gkg770 14500829 PMC206470

[B20] Haas B. J. Salzberg S. L. Zhu W. Pertea M. Allen J. E. Orvis J. (2008). Automated eukaryotic gene structure annotation using EVidenceModeler and the program to assemble spliced alignments. Genome Biol. 9 (1), R7. 10.1186/gb-2008-9-1-r7 18190707 PMC2395244

[B21] Huang Y. Wang Z. Zha S. Wang Y. Jiang W. Liao Y. (2016). De novo transcriptome and expression profile analysis to reveal genes and pathways potentially involved in cantharidin biosynthesis in the blister beetle mylabris cichorii. PloS One 11 (1). 10.1371/journal.pone.0146953 PMC4709229 26752526

[B22] Hong A. Y. Stoltz B. M. (2014). Biosynthesis and chemical synthesis of presilphiperfolanol natural products. Angew. Chem. Int. Ed. Engl. 235, 326–332. 10.1002/anie.201309494 PMC4334158 24771653

[B23] Jiang M. Lu S. Zhang Y. (2017). The potential organ involved in cantharidin biosynthesis in Epicauta chinensis Laporte (Coleoptera: Meloidae). J. Insect Sci. 17 (2). 10.1093/jisesa/iex021 PMC5633858 28423415

[B24] Jiang M. Lü S. Zhang Y. (2017). Characterization of juvenile hormone related genes regulating cantharidin biosynthesis in epicauta chinensis. Sci. Rep. 7 (1), 2308. 10.1038/s41598-017-02393-w 28536442 PMC5442126

[B25] Jiang M. Lue S.-M. Qi Z.-Y. Zhang Y.-L. (2019). Characterized cantharidin distribution and related gene expression patterns in tissues of blister beetles, Epicauta chinensis. Insect Sci. 26 (2), 240–250. 10.1111/1744-7917.12512 28745022

[B26] Kadioglu O. Kermani N. S. Kelter G. Schumacher U. Fiebig H.-H. Greten H. J. (2014). Pharmacogenomics of cantharidin in tumor cells. Biochem. Pharmacol. 87 (3), 399–409. 10.1016/j.bcp.2013.10.025 24231507

[B27] Karras D. J. Farrell S. E. Harrigan R. A. Henretig F. M. Gealt L. (1996). Poisoning from "Spanish fly" (cantharidin). Am. J. Emergency Med. 14 (5), 478–483. 10.1016/S0735-6757(96)90158-8 8765116

[B28] Katoh K. (2002). MAFFT: a novel method for rapid multiple sequence alignment based on fast Fourier transform. Nucleic Acids Res. 30 (14), 3059–3066. 10.1093/nar/gkf436 12136088 PMC135756

[B29] Keeling C. I. Yuen M. M. S. Liao N. Y. Docking T. R. Chan S. K. Taylor G. A. (2013). Draft genome of the mountain pine beetle, Dendroctonus ponderosae Hopkins, a major forest pest. Genome Biol. 14 (3), R27. 10.1186/gb-2013-14-3-r27 23537049 PMC4053930

[B30] Keller O. Kollmar M. Stanke M. Waack S. (2011). A novel hybrid gene prediction method employing protein multiple sequence alignments. Bioinformatics 27 (6), 757–763. 10.1093/bioinformatics/btr010 21216780

[B31] Koren S. Walenz B. P. Berlin K. Miller J. R. Bergman N. H. Phillippy A. M. (2017). Canu: scalable and accurate long-read assembly via adaptive k-mer weighting and repeat separation. Genome Res. 27 (5), 722–736. 10.1101/gr.215087.116 28298431 PMC5411767

[B32] Li W. Xie L. Chen Z. Zhu Y. Sun Y. Miao Y. (2010). Cantharidin, a potent and selective PP2A inhibitor, induces an oxidative stress-independent growth inhibition of pancreatic cancer cells through G2/M cell-cycle arrest and apoptosis. Cancer Sci. 101 (5), 1226–1233. 10.1111/j.1349-7006.2010.01523.x 20331621 PMC11158714

[B33] Li W. Kondratowicz B. McWilliam H. Nauche S. Lopez R. (2013). The Annotation-enriched non-redundant patent sequence databases. Database. bat005. 10.1093/database/bat005 23396323 PMC3568390

[B34] Li H-c Xia Z-h Chen Y-f Yang F. Feng W. Cai H. (2017). Cantharidin inhibits the growth of triple-negative breast cancer cells by suppressing autophagy and inducing apoptosis *in vitro* and *in vivo* . Cell. Physiol. Biochem. 43 (5), 1829–1840. 10.1159/000484069 29050003

[B35] Li H. (2018). Minimap2: pairwise alignment for nucleotide sequences. Bioinformatics 34 (18), 3094–3100. 10.1093/bioinformatics/bty191 29750242 PMC6137996

[B36] Liu D. Chen Z. (2009). The effects of Cantharidin and Cantharidin derivates on tumour cells. Anti-Cancer Agents In Med. Chem. 9 (4), 392–396. 10.2174/1871520610909040392 19442040

[B37] Liu B. Shi Y. Yuan J. Hu X. Zhang H. Li N. (2009). Estimation of genomic characteristics by analyzing k-mer frequency in de novo genome projects. Quant. Biol. arXiv:1308.2012.

[B38] Lobo I. (2012). Basic local alignment search tool (BLAST). J. Mol. Biol. 215 (3), 403–410. 10.1006/jmbi.1990.9999 2231712

[B39] Lu S. Jiang M. Huo T. Li X. Zhang Y. (2016). 3-hydroxy-3-methyl glutaryl coenzyme a reductase: an essential actor in the biosynthesis of cantharidin in the blister beetle Epicauta chinensis Laporte. Insect Mol. Biol. 25 (1), 58–71. 10.1111/imb.12198 26566751

[B40] Luan J. Duan H. Liu Q. Yagasaki K. Zhang G. (2010). Inhibitory effects of norcantharidin against human lung cancer cell growth and migration. Cytotechnology 62 (4), 349–355. 10.1007/s10616-009-9250-8 20087654 PMC2978303

[B41] Manekar S. C. Sathe S. R. (2018). A benchmark study of k-mer counting methods for high-throughput sequencing. Gigascience 7 (12), giy125. 10.1093/gigascience/giy125 PMC6280066 30346548

[B42] McCormick J. P. Carrel J. E. Doom J. P. (1986). Origin of oxygen atoms in cantharidin biosynthesized by beetles. J. Am. Chem. Soc. 108 (25), 8071–8074. 10.1021/ja00285a032

[B43] Mckenna D. D. Scully E. D. Pauchet Y. Hoover K. Kirsch R. Geib S. M. (2016). Genome of the Asian longhorned beetle (Anoplophora glabripennis), a globally significant invasive species, reveals key functional and evolutionary innovations at the beetle–plant interface. Genome Biol. 17 (1), 227. 10.1186/s13059-016-1088-8 27832824 PMC5105290

[B44] Moed L. Shwayder T. A. Chang M. W. (2001). Cantharidin revisited: a blistering defense of an ancient medicine. Arch. Dermatol. 137 (10), 1357–1360. 10.1001/archderm.137.10.1357 11594862

[B45] Moriya Y. Itoh M. Okuda S. Yoshizawa A. C. Kanehisa M. (2007). KAAS: an automatic genome annotation and pathway reconstruction server. Nucleic Acids Res. 35 (Web Server issue), W182–W185. 10.1093/nar/gkm321 17526522 PMC1933193

[B46] Pertea M. Pertea G. M. Antonescu C. M. Chang T.-C. Mendell J. T. Salzberg S. L. (2015). StringTie enables improved reconstruction of a transcriptome from RNA-seq reads. Nat. Biotechnol. 33 (3), 290–295. 10.1038/nbt.3122 25690850 PMC4643835

[B47] Price A. L. Jones N. C. Pevzner P. A. (2005). De novo identification of repeat families in large genomes. Bioinf. (Oxford England) 21 Suppl 1, i351–i358. 10.1093/bioinformatics/bti1018 15961478

[B48] Rice P. Longden I. Bleasby A. (2000). EMBOSS: the European molecular biology open software suite. Trends in Genet. 16 (6), 276–277. 10.1016/S0168-9525(00)02024-2 10827456

[B49] Richard T. Michael P. Ellen D. M. Jacob L. (2014). Cantharidin: a comprehensive review of the clinical literature. Dermatol. Online J. 20 (6). 24945640

[B50] Richards S. Gibbs R. A. Weinstock G. M. Brown S. J. Denell R. Beeman R. W. (2008). The genome of the model beetle and pest Tribolium castaneum. Nat. 452 (7190), 949–955. 10.1038/nature06784 18362917

[B51] Schoville S. D. Chen Y. H. Andersson M. N. Benoit J. B. Bhandari A. Bowsher J. H. (2018). A model species for agricultural pest genomics: the genome of the Colorado potato beetle, Leptinotarsa decemlineata (Coleoptera: Chrysomelidae). Sci. Rep. 8 (1), 1931. 10.1038/s41598-018-20154-1 29386578 PMC5792627

[B52] Silverberg N. B. Sidbury R. Mancini A. J. (2000). Childhood molluscum contagiosum: experience with cantharidin therapy in 300 patients. J. Am. Acad. Dermatol. 43 (3), 503–507. 10.1067/mjd.2000.106370 10954663

[B53] Simao F. A. Waterhouse R. M. Ioannidis P. Kriventseva E. V. Zdobnov E. M. (2015). BUSCO: assessing genome assembly and annotation completeness with single-copy orthologs. Bioinformatics 31 (19), 3210–3212. 10.1093/bioinformatics/btv351 26059717

[B54] Su C. C. Lee K. I. Chen M. K. Kuo C. Y. Tang C. H. Liu S. H. (2016). Cantharidin induced oral squamous cell carcinoma cell apoptosis via the JNK-regulated mitochondria and endoplasmic reticulum stress-related signaling pathways. PloS One 11 (12), e0168095. 10.1371/journal.pone.0168095 27930712 PMC5145211

[B55] Tagwireyi D. Ball D. E. Loga P. J. Moyo S. (2000). Cantharidin poisoning due to “Blister beetle” ingestion. Toxicon : Off. J. Int. Soc. Toxinology 38 (12), 1865–1869. 10.1016/S0041-0101(00)00093-3 10858524

[B56] Trapnell C. Roberts A. Goff L. Pertea G. Kim D. Kelley D. R. (2012). Differential gene and transcript expression analysis of RNA-seq experiments with TopHat and Cufflinks. Nat. Protoc. 7 (3), 562–578. 10.1038/nprot.2012.016 22383036 PMC3334321

[B57] UniProt Consortium (2010). The universal protein resource (UniProt) in 2010. Nucleic Acids Res. 38 (Database issue), D142–D148. 10.1093/nar/gkp846 19843607 PMC2808944

[B58] Walker B. J. Abeel T. Shea T. Priest M. Abouelliel A. Sakthikumar S. (2014). Pilon: an integrated tool for comprehensive microbial variant detection and genome assembly improvement. PloS One 9 (11), e112963. 10.1371/journal.pone.0112963 25409509 PMC4237348

[B59] Wang G. S. (1989). Medical uses of mylabris in ancient China and recent studies. J. Ethnopharmacol. 26 (2), 147–162. 10.1016/0378-8741(89)90062-7 2689797

[B60] Wouter D. C. Arne D. R. Tim D. P. Svenn D. H. Peter D. R. Mojca S. (2019). Structural variants identified by Oxford Nanopore PromethION sequencing of the human genome. Genome Res. (7), 1178–1187. 10.1101/gr.244939.118 31186302 PMC6633254

[B61] Wu Y.-M. Li J. Chen X.-S. (2018). Draft genomes of two blister beetles Hycleus cichorii and Hycleus phaleratus. Gigascience 7 (3). 10.1093/gigascience/giy006 PMC5905561 29444297

[B62] Xiao C.-L. Chen Y. Xie S.-Q. Chen K.-N. Wang Y. Han Y. (2017). MECAT: fast mapping, error correction, and de novo assembly for single-molecule sequencing reads. Nat. Methods 14 (11), 1072–1074. 10.1038/nmeth.4432 28945707

[B63] Xu Z. Wang H. (2007). LTR_FINDER: an efficient tool for the prediction of full-length LTR retrotransposons. Nucleic Acids Res. 35 (Web Server issue), W265–W268. 10.1093/nar/gkm286 17485477 PMC1933203

[B64] Yang Z. Rannala B. (2006). Bayesian estimation of species divergence times under a molecular clock using multiple fossil calibrations with soft bounds. Mol. Biol. Evol. 23 (1), 212–226. 10.1093/molbev/msj024 16177230

[B65] Yin Y. P. Jin G. X. (2010). Biosynthesis,transfer and biological function of cantharidin in blister beetles(Coleoptera:Meloidae). Acta Entomol. Sin. 53 (11), 1305–1313. 10.1016/S1002-0721(10)60377-8

[B66] Zhang W. Ma Y.-Z. Song L. Wang C.-H. Qi T.-G. Shao G.-R. (2014). Effect of Cantharidins in Chemotherapy for Hepatoma: a retrospective cohort study. Am. J. Chin. Med. 42 (3), 561–567. 10.1142/S0192415X14500360 24871651

