# Supplementary material for: Draft Genome of a Blister Beetle Mylabris aulica
Source: Front Genet. 2020 Jan 8;10:1281. doi: 10.3389/fgene.2019.01281 (PMC6972506; doi:10.3389/fgene.2019.01281)
Supplement: Supplementary file 1 [file Table_1.docx]

Table S1: The identified of repeat elements, total 153144758 bp sites were identified.

The Repeat sequences classified according to different types.

| DNA: |  |  |  |
| --- | --- | --- | --- |
|  | Academ | 44 | 163593 |
|  | Academ-1 | 9 | 16469 |
|  | CMC-Chapaev-3 | 63 | 42760 |
|  | CMC-EnSpm | 1187 | 1100757 |
|  | CMC-Transib | 11 | 10189 |
|  | Crypton | 271 | 156397 |
|  | Crypton-H | 4 | 17671 |
|  | Crypton-I | 668 | 113609 |
|  | Dada | 2 | 38166 |
|  | Ginger | 32 | 85568 |
|  | Kolobok | 118 | 126689 |
|  | Kolobok-E | 420 | 229723 |
|  | Kolobok-Hydra | 224 | 107579 |
|  | MULE-MuDR | 79 | 80305 |
|  | Maverick | 1723 | 3534830 |
|  | Merlin | 116 | 91147 |
|  | MuLE-MuDR | 108 | 164023 |
|  | P | 1117 | 1223352 |
|  | PIF-Harbinger | 802 | 658944 |
|  | PIF-ISL2EU | 346 | 270007 |
|  | PIF-Spy | 22 | 29054 |
|  | PiggyBac | 535 | 566028 |
|  | PiggyBac? | 13 | 2718 |
|  | Sola | 37 | 71716 |
|  | Sola-1 | 32 | 38043 |
|  | Sola-2 | 46 | 79830 |
|  | TcMar-Fot1 | 138 | 318643 |
|  | TcMar-ISRm11 | 29 | 38833 |
|  | TcMar-Mariner | 755 | 410046 |
|  | TcMar-Pogo | 157 | 254881 |
|  | TcMar-Tc1 | 3879 | 2033675 |
|  | TcMar-Tc1? | 1 | 527 |
|  | TcMar-Tc4 | 2296 | 737801 |
|  | TcMar-Tigger | 261 | 153922 |
|  | TcMar-m44 | 1014 | 532200 |
|  | Zisupton | 42 | 9634 |
|  | hAT | 142 | 48205 |
|  | hAT-Ac | 765 | 354694 |
|  | hAT-Blackjack | 124 | 202716 |
|  | hAT-Charlie | 287 | 180853 |
|  | hAT-Tag1 | 363 | 194730 |
|  | hAT-Tip100 | 415 | 492816 |
|  | hAT-hAT19 | 19 | 10927 |
|  | hAT-hATm | 2 | 3068 |
|  | hAT-hATx | 22 | 52831 |
| LINE: |  |  |  |
|  | CR1 | 4 | 10213 |
|  | Dong-R4 | 206 | 338993 |
|  | I | 763 | 590982 |
|  | I-Jockey | 80 | 254326 |
|  | I-Nimb | 250 | 268413 |
|  | Jockey | 379 | 339496 |
|  | L1 | 42 | 134520 |
|  | L2 | 28 | 171622 |
|  | LOA | 840 | 382701 |
|  | Penelope | 1209 | 1276529 |
|  | R1 | 1232 | 974634 |
|  | R1-LOA | 173 | 66553 |
|  | R2 | 234 | 289607 |
|  | R2-NeSL | 20 | 22505 |
|  | RTE-BovB | 293 | 91268 |
|  | RTE-X | 43 | 26093 |
|  | Tad1 | 218 | 39821 |
| LTR: |  |  |  |
|  | Copia | 225 | 228379 |
|  | ERV1 | 61 | 47214 |
|  | Gypsy | 5851 | 6138858 |
|  | Ngaro | 16 | 27781 |
|  | Pao | 846 | 1451996 |
| SINE: |  |  |  |
|  | tRNA | 10 | 703 |
|  | tRNA-Deu-RTE | 8 | 757 |
|  | tRNA-I | 3 | 89 |
|  | tRNA-RTE | 21 | 1505 |
| ARTEFACT | 1 | 423 |  |
| Low_complexity | 17567 | 1099440 |  |
| RNA | 274 | 30395 |  |
| Satellite | 730 | 285726 |  |
| Simple_repeat | 160397 | 30552633 |  |
| Unknown | 108500 | 91421577 |  |
| rRNA | 499 | 634512 |  |
